# *Blouch*: Bayesian Linear Ornstein-Uhlenbeck Models for Comparative Hypotheses

**DOI:** 10.1093/sysbio/syae044

**Published:** 2024-09-02

**Authors:** Mark Grabowski

**Affiliations:** Research Centre for Evolutionary Anthropology and Palaeocology, School of Biological and Environmental Sciences, Liverpool John Moores University, James Parson Building, 3 Byrom Street, Liverpool L3 3AF, UK; Department of Biosciences, CEES, University of Oslo, Blinderen, PB 1066, 0316 Oslo, Norway

**Keywords:** adaptation, Bayesian, Ornstein-Uhlenbeck, phylogenetic comparative methods, *Stan*

## Abstract

Relationships among species in the tree of life can complicate comparative methods and testing adaptive hypotheses. Models based on the Ornstein-Uhlenbeck process permit hypotheses about adaptation to be tested by allowing traits to either evolve toward fixed adaptive optima (e.g., regimes or niches) or track continuously changing optima that can be influenced by other traits. These models allow estimation of the effects of both adaptation and phylogenetic inertia—resistance to adaptation due to any source—on trait evolution, an approach known as the “adaptation-inertia” framework. However, previous applications of this framework, and most approaches suggested to deal with the issue of species non-independence, are based on a maximum likelihood approach, and thus it is difficult to include information based on prior biological knowledge in the analysis, which can affect resulting inferences. Here, I present *Blouch*, (Bayesian Linear Ornstein-Uhlenbeck Models for Comparative Hypotheses), which fits allometric and adaptive models of continuous trait evolution in a Bayesian framework based on fixed or continuous predictors and incorporates measurement error. I first briefly discuss the models implemented in *Blouch*, and then the new applications for these models provided by a Bayesian framework. This includes the advantages of assigning biologically meaningful priors when compared to non-Bayesian approaches, allowing for varying effects (intercepts and slopes), and multilevel modeling. Validations on simulated data show good performance in recovering the true evolutionary parameters for all models. To demonstrate the workflow of *Blouch* on an empirical dataset, I test the hypothesis that the relatively larger antlers of larger-bodied deer are the result of more intense sexual selection that comes along with their tendency to live in larger breeding groups. While results show that larger-bodied deer that live in larger breeding groups have relatively larger antlers, deer living in the smallest groups appear to have a different and steeper scaling pattern of antler size to body size than other groups. These results are contrary to previous findings and may argue that a different type of sexual selection or other selective pressures govern optimum antler size in the smallest breeding groups.

Since Felsenstein (1985), it has been widely recognized that phylogenetic relatedness among species can complicate comparative studies and testing of adaptive hypotheses (Hansen 1997; Harvey and Pagel 1998; Butler and King 2004; Hansen and Orzack 2005; Hansen and Bartoszek 2012). A wide variety of approaches have been suggested to deal with the issue of species non-independence (see O’Meara 2012 and references within), though the pattern of evolution will dictate whether such approaches are needed (Westoby et al. 1995; Ricklefs and Starck 1996; Björklund 1997; Hansen and Orzack 2005; Hansen et al. 2008; Uyeda et al. 2018), as originally argued by Felsenstein (1985). Hansen (1997) introduced a model of adaptive evolution based on the Ornstein-Uhlenbeck process. In this model, hypotheses about adaptation can be explored by allowing traits to either evolve toward fixed adaptive optima, where lineages have been assigned a priori to a particular niche (see Uyeda and Harmon 2014 for more on this point) or track continuously changing optima that can be influenced by other traits (Hansen et al. 2008). A key motivation for the use of Ornstein-Uhlenbeck models in phylogenetic comparative studies is the ability to estimate the effects of both adaptation and phylogenetic inertia—resistance to adaptation due to any source—on trait evolution, and the approach has been coined the “adaptation-inertia” framework (Hansen et al. 2008).

Most previous approaches to modeling trait evolution toward optima influenced by continuous or categorical predictors are based on maximum likelihood approaches (e.g., Butler and King 2004; Bartoszek et al. 2012; Beaulieu et al. 2012; Hansen and Bartoszek 2012; Pienaar et al. 2013), with some notable exceptions (Uyeda and Harmon 2014; Clavel et al. 2015; Höhna et al. 2016; Ross et al. 2016; Uyeda et al. 2017; Bastide et al. 2021). This includes the current version of *Slouch* (Stochastic Linear Ornstein-Uhlenbeck Comparative Hypotheses) (Hansen et al. 2008; Kopperud et al. 2020), which allows for the testing of adaptive hypotheses using both categorical (i.e., adaptive regimes) and continuous predictor data, and the multivariate implementation of the OU model, *mvSlouch* (Bartoszek et al. 2012, 2023).

Complementing Uyeda and Harmon (2014), taking a Bayesian approach to phylogenetic comparative methods has several distinct advantages that are embedded in Bayesian statistics. First, if we have prior information about biologically relevant parameters, Bayesian statistics provide a natural way to incorporate it. For example, non-Bayesian approaches implicitly assume that prior distributions are flat by design, which may or may not be a realistic assumption. Placing informative priors on parameters allows us to restrict parameter space to regions to test specific hypotheses or that can be judged meaningful in a particular biological context (Uyeda and Harmon 2014), such as quantitative genetic models (Lande 1976; Uyeda and Harmon 2014). Priors can also be used to remedy previously identified issues in fitting evolutionary models to phylogenetically structured data (Ho and Ané 2014; Cornuault 2022) such as likelihood ridges due to correlated parameters (Uyeda and Harmon 2014). Second, Bayesian methods provide improved tools to observe and ways to estimate uncertainty. The parameter estimates themselves may not be better, or more accurate, than those produced in a maximum likelihood framework, but we have a clearer understanding of uncertainty in the Bayesian framework (McElreath 2020). Third, taking a Bayesian approach provides methods to easily incorporate uncertainty at multiple levels via multilevel or hierarchical models (Gelman and Hill 2006). For example, incorporating variation within and among species in the allometric relationship among traits, or using partial pooling—pooling information across groups—to produce more accurate estimates of optima. Fourth, the parameter estimates themselves are arguably more understandable and do not rely on assumptions about repeated sampling. For example, the phylogenetic half-life, *t*_1/2_, quantifies the average time for the trait to evolve half the distance from the ancestral state value to the primary optimum. In a maximum likelihood approach, uncertainty around the point estimate is often quantified by reporting 2-unit support surfaces, the range of parameters for which the log-likelihood is no more than two units away from the maximum likelihood (Hansen et al. 2008). Using a Bayesian approach, an *n*% compatibility interval or region (CI) is the interval within which the unobserved parameter values fall with *n* probability. This is arguably a more intuitive metric than the alternative, which could aid in biological interpretation. Finally, the posterior distribution of parameters can easily be incorporated as data in downstream analyses.

Here, I present *Blouch* (Bayesian Linear Ornstein-Uhlenbeck models for Comparative Hypotheses), a package for studying adaptive evolution using Ornstein-Uhlenbeck models. *Blouch* builds on the R package *Slouch* (Hansen et al. 2008; Kopperud et al. 2020) but places its approach within a Bayesian framework and implements several features not currently available in existing phylogenetic comparative methods. While the front-end component of *Blouch* is written in R (R Core Team 2024), the nuts and bolts are written in the language Stan (Carpenter et al. 2017), which allows estimation of Bayesian models using Markov chain Monte Carlo (MCMC) methods based on the Hamiltonian Monte Carlo sampler. Hamiltonian Monte Carlo offers advantages over Metropolis-Hastings and Gibbs samplers as it allows for more efficient sampling of complex models, including needing less samples to describe the posterior distribution, reduces autocorrelation, and contains internal checks of efficiency and accuracy (Nishio and Arakawa 2019).

I first briefly discuss the models implemented in *Blouch*, and then introduce the use and meaning of prior distributions for the estimated parameters within a biologically meaningful framework. I then present the new features that are introduced in this package. Extensive validation of *Blouch* is included in the Supplementary Material available in the Dryad data repository (https://doi.org/10.5061/dryad.rv15dv4dx) and the project GitHub site (see below), but here I use a simulated dataset to compare implemented models and to demonstrate the workflow. Finally, to demonstrate its use on an empirical dataset, I test the hypothesis that the relatively larger antlers of larger-bodied deer are the result of more intense sexual selection that comes along with their tendency to live in larger breeding groups.

## Overview of *Blouch*

### Implemented Models

Following Hansen (1997) and Hansen et al. (2008), *Blouch* models evolution in the response trait as an Ornstein-Uhlenbeck process around a primary optimal state that is a function of the predictor variables, which can be mapped as (fixed) adaptive regimes on the phylogeny (i.e., multi-optima models; Hansen 1997; Butler and King 2004) or modeled as evolving following a Brownian-motion process (Hansen et al. 2008). For continuous predictors, *Blouch* follows the approach of Grabowski et al. (2016; 2023) in implementing both the model of adaptive evolution introduced by Hansen et al. (2008) and the model of constrained evolution (termed the direct effect model here as in Grabowski et al. 2023) introduced in Hansen and Bartoszek (2012; see also Grabowski et al. 2016, 2023b), which is appropriate for testing allometric hypotheses.

Below is the simple stochastic multi-optima model of the Ornstein-Uhlenbeck process to aid in the subsequent discussions. Following Hansen (1997), *Blouch* models the evolution of a response variable as an Ornstein-Uhlenbeck process toward a primary optimal state that is a function of categorical predictor variables (e.g., social systems) mapped as regimes on the phylogeny, a relationship expressed as the stochastic differential equation:


$$\begin{array}{l} dy=-\alpha \left(y-\theta (z\right))dt+\sigma_{y}dB_{y} \end{array}$$

where *dy* is changes in the response variable, *y*, over a small time interval, *dt*, α is the rate of adaptation of *y* toward the optimum θ, modeled as a function of the categorical predictors mapped on the phylogeny, *z*, and can be expressed as the phylogenetic half-life, $t_{1/2}=\ln\left(2\right)/\alpha$, *dB* is a white-noise process (independent normally distributed random changes with mean 0 and unit variance) and $\sigma_{y}$ is the standard deviation of the random changes, which can be expressed as the stationary variance, $v=\sigma_{y}^{2}/\left(2\alpha \right)$, the equilibrium variance of an Ornstein-Uhlenbeck process evolving around a stationary selective optimum, θ.

Further details on implementations of Ornstein-Uhlenbeck models are included in the Supplementary Materials, with new implementations described below.

### Use and Meaning of Priors in the Adaptation-Inertia Framework

As suggested by Ho and Ané (2014) and recently explored by Cornuault (2022) (see also Uyeda and Harmon 2014), priors for parameters in Bayesian approaches to fitting Ornstein-Uhlenbeck models such as the rate of adaptation, α, can affect posterior estimates for a variety of model parameters due to interactions between parameters. For example, Cornuault (2022) showed that using a prior concentrated at one or the other extreme values of α, translating to nearly instantaneous adaptation or a Brownian motion process, affects the marginal posterior distribution of α, regardless of whether the data was generated under Brownian motion or instantaneous adaptation. These extreme priors on α also affect the standard deviation of random changes, σ, and the estimated selective optima, θ, though in complex ways that vary across the parameters (Cornuault 2022). However, as stressed by Cornuault (2022) and expanded on by Grabowski et al. (2023b), interpretation of parameters in Ornstein-Uhlenbeck models is fundamentally important for valid inference, such as by transforming parameters into biologically understandable units (e.g., from time^−1^ for α to time for *t*_*1/2*_) and setting reasonable and biologically informed values on priors as discussed below.

*Blouch* allows prior probability distributions to be placed on parameters in meaningful units for macroevolutionary models, including the half-life (*t*_*1/2*_), stationary variance (*v*), selective optima (θ), and the optimal and direct effect slopes (Hansen et al. 2008). Below, I discuss the interpretation of each parameter and provide suggestions for setting appropriate prior distributions. Note that for any analyses, prior predictive simulations should always be conducted to determine sensible values.

#### Phylogenetic half-life (*t*_*1/2*_).

The degree of phylogenetic correlation in the model residuals is estimated from the data and measured by the phylogenetic half-life (*t*_*1/2*_), which estimates on average how long it takes to evolve half-way from the ancestral state to a new optimum given a regime shift. Because *Blouch* scales tree height to one for analyses as part of its setup, extreme values on priors for *t*_*1/2*_ are equivalent to whether one assumes adaptation occurs quickly, with very small values of *t*_*1/2*_, or increasingly slowly, with *t*_*1/2*_ values >>> 1 (tree height), suggesting the data or residuals are increasingly following a Brownian motion process. Though this may vary depending on the research question, setting an initial prior that includes both nearly instantaneous adaptation and between one to multiple tree heights is a conservative approach. For example, setting an initial prior on *t*_*1/2*_ that is a Lognormal distribution where the lower 10% is equivalent to 10% of tree height, and the upper 10% starts at one to multiple times the height of the tree (see Grabowski et al. 2023a for a similar setup). In the empirical Cervidae example given below, the tree height is 14 Ma. Following this guide, a reasonable initial prior would have 10% of the values of the distribution less than about ~ 1.4 Ma and about 10% of the values greater than 14 Ma when transformed back to the original unscaled tree height.

#### Stationary variance (v).

The stationary variance estimates the variance around a selective optimum at equilibrium—the expected among-species variance for species that evolved for a long time in a constant niche (Hansen 2008). It is reasonable to assume that the current among-species variance in the response variable might give an indication on the prior for *v*. Thus, a noninformative prior for *v* could be a uniform prior with limits from zero to about 4 times this variance (Gelman et al. 2013) but using an exponential or half-*t* distribution can permit prior information to be included more easily and could produce more biologically realistic results. Given a multi-level model such as introduced below with a relatively small number of groups (i.e., regimes or niches), a uniform prior may lead to high estimates of variance parameters, and a distribution from the half-*t* family of prior distributions is recommended, such as a half-Cauchy (Gelman 2006). It is important to note that, like any parameter, inspection of the posterior distribution of *v* may provide new knowledge as to the appropriateness of this prior distribution for further analysis (Gelman 2006). In the validation and simulation steps below, the true values for *v* are set at a small number (0.01), which may be less realistic for models with long half-lives as the two parameters are correlated (Hansen 2008) but allow model performance given changing values of *t*_*1/2*_ to be determined. For these simulations, an exponential prior was used with relatively large rate values after inspecting the posterior distributions.

#### Primary optima in multi-optima models (θ).

In the case of multi-optima models, optima are estimated based on the observed values of the response variable (Y) and two or more selective regimes placed on a phylogeny. A reasonable prior would thus be centered at the mean value of Y, and a standard deviation informed by the biological hypotheses being tested. For example, one approach would be to use a normal distribution centered on the mean Y with a standard deviation of 1 if the hypothesized maximum difference between the estimated optima was ~ 2 units. This prior allows for 95% of the probability to be two units less and two units more than the mean, allowing for an informed but reasonably wide prior.

#### Optimal and evolutionary slopes.

In the adaptation model, the response variable evolves according to an Ornstein-Uhlenbeck process toward an optimal state that is modeled as a function of the predictor variable. This model estimates the optimal and evolutionary regressions, the former the relationship between the response and predictor that would be achieved if the response was able to adapt fast enough to perfectly track changes in the predictor—the best fit of *θ* on *X*—the latter is the generalized least square regression of the response on the predictor and is influenced by both adaptation and inertia—the best fit of *Y* on *X* (Hansen et al. 2008). The optimum here is now the optimal regression line, and the half-life is thus an average estimate of how long it takes for a maladapted species evolve half the distance to the optimum. If adaptation is nearly instantaneous, the optimal and evolutionary slope will converge, with the latter diverging from the former whenever there is a lag in adaptation—quantified by *t*_*1/2*_. For a phylogeny scaled to unit height, a short half-life (a small percentage of the length of the phylogeny) means that the response variable rapidly adapts toward θ. A very long half-life (i.e., ~ multiple times the length of the phylogeny) means the model converges on Brownian motion. As instantaneous adaptation is akin to no phylogenetic effect, a reasonable prior for the optimal slope might be a normal distribution centered on the ordinary least squares slope with a standard deviation based on prior predictive simulations compared to the spread of the data. The evolutionary slope is the product of the optimal slope and ρ, the phylogenetic correction factor (Hansen et al. 2008), which provides a metric to estimate the effects of phylogenetic inertia. A value of ρ=1 would mean there are no effects and the evolutionary slope and the optimal slope converge, with decreasing values of ρ leading to decreasing values of the evolutionary slope. Based on this justification, the prior on the evolutionary slope might be a normal distribution centered around this product with a scale informed by prior predictive simulations. On the other hand, as the evolutionary slope is directly related to this product, a deterministic definition might be more appropriate. *Blouch* can be coded to include both formulations but here the results shown are based on the deterministic definition.

#### Direct effect slope.

In the direct effect model, changes in the continuous predictor are associated with an immediate correlated response in the response variable, which can be used to test for allometric constraints (Hansen and Bartoszek 2012; Grabowski et al. 2016, 2023a). Biologically, the direct effect model can be thought of as a single-optimum model where the optimum is along the slope of the regression line, with $t_{1/2}$ measuring the phylogenetic inertia of the model residuals around that optimum, and *v* measuring the stationary variance predicted from the model. Note that there is no empirical reason we expect to have correlated residuals in this model—these effects are not built into the model itself—and hence no reason to expect $t_{1/2}$ > 0. Correlated residuals as an empirical finding may arise from a variety of influences (Hansen and Bartoszek 2012). For example, we might expect that the species residuals from the model are being pulled toward the regression line; for example, selection might lead to species with greater and greater values of *Y* for a given *X* to be less fit, but this is not built into the model. As changes in the predictor (X) lead to immediate changes in the response variable (Y) mediated by the direct effect slope parameter *b*, we expect the intercept and slope parameters to be similar to an ordinary least squares regression. Hence, reasonable priors on the intercept and the slope would be centered on these values, with standard deviations based on prior predictive simulations compared to the spread of the data as for the adaptive model.

### New Features

#### Multilevel multi-optima and varying effects models.

For multi-optima models or models that are a combination of multi-optima and continuous predictors, *Blouch* can fit multilevel models (also called hierarchical models) that allow information to be shared across regimes and permit different types of varying effects models (Gelman 2005; Gelman et al. 2013; McElreath 2020) where the intercept and slope terms are allowed to differ from regime to regime. In multilevel models, the estimated parameters are viewed as a sample from a common population distribution of parameters, which have their own parameters known as hyperparameters. Here the priors on the parameters are adaptively learned from the data, rather than being chosen beforehand. For example, the regime optima, $\theta_{reg\left[i\right]},\;$for a given species *i*, could be modeled as $\theta_{reg\left[i\right]}=Normal\left(\bar{\theta },\;\sigma \right)$, with $\bar{\theta }$ the hyperparameter and is the prior for the average regime and σ the standard deviation among the regimes. By learning about each regime simultaneously across the population of regimes, multilevel models allow information to be shared across these groupings, based on the variation among the regimes. Known as partial pooling, this facility can improve accuracy of the parameter estimates. Multilevel models can provide improved estimates for analyses with imbalance in sampling, provide estimates of variation within and among groups, and balance overfitting—where models learn too much from the data—and underfitting—where models learn too little from the data (Gelman and Hill 2006; McElreath 2020).

Below is the mathematical model definition for the simplest multilevel model that allows partial pooling across regimes and varying intercepts across regimes for the multi-optima model:


$$\begin{array}{l} Y_{i}∼MVNormal\left(u_{i},V\right) \end{array}$$

$$\begin{array}{l} u_{i}=dmX_{i}\theta_{reg\left[i\right]} \end{array}$$

$$\begin{array}{l} dmX_{i}=f\left(t_{1/2},T\right) \end{array}$$

$$\begin{array}{l} \theta_{reg\left[i\right]}=Normal\left(\bar{\theta },\sigma \right) \end{array}$$

$$\begin{array}{l} \bar{\theta }∼Normal\left(0,1\right) \end{array}$$

$$\begin{array}{l} \sigma ∼Exponential\left(5\right) \end{array}$$

$$\begin{array}{l} V=g\left(t_{1/2},v,T\right) \end{array}$$

$$\begin{array}{l} t_{1/2}∼logNormal\left(log\left(0.25\right),0.25\right) \end{array}$$

$$\begin{array}{l} v∼Exponential\left(20\right) \end{array}$$

where $Y_{i}$ is the observed species values with a multivariate Gaussian distribution with mean $u_{i}$ and variance/covariance matrix V, $u_{i}$ is defined as the product of $dmX_{i}$ and the optima $\theta_{reg\left[i\right]}$, with $dmX_{i}$ a design matrix derived in Hansen (1997) with the number of rows equal to the number of species and number of columns equal to the number of θ, and elements giving the sum of weights of segments on the tree *T* where the optima was $\theta_{reg\left[i\right]}$ and is a the result of a function of the phylogenetic half-life, $t_{1/2}$_,_ and the phylogeny, *T*. $\theta_{reg\left[i\right]}$ is a vector with each element the optimal value for a given regime and has a normal distribution centered on $\bar{\theta }$ with standard deviation σ, *V* is the variance/covariance matrix defined as in Hansen (1997) and is the result of a function of $t_{1/2}$, the stationary variance, v, and *T*. The priors above are examples that match those used in the simulation study below.

For multilevel multi-optima models combined with direct effect and/or adaptive predictors and varying effects, *Blouch* allows for covariance between optima/intercepts and slopes using a 2-dimensional multivariate Gaussian distribution. Below is the simplest multilevel multi-optima direct effect model that allows partial pooling across the parameters across regimes. This model includes varying intercepts across the regimes but also varying slopes for the direct effect predictor, *X*, and includes a joint population of varying intercepts and slopes.


$$\begin{array}{l} Y_{i}∼MVNormal\left(u_{i},V\right) \end{array}$$

$$\begin{array}{l} u_{i}=dmX_{i}\theta_{reg\left[i\right]}+X_{i}\beta_{reg\left[i\right]} \end{array}$$

$$\begin{array}{l} \left[\theta_{reg\left[i\right]},\beta_{reg\left[i\right]}\right]∼MVNormal\left(\left[\bar{\theta },\bar{\beta }\right],R,\left[\sigma_{\theta },\sigma_{\beta }\right]\right) \end{array}$$

$$\begin{array}{l} dmX_{i}=f\;\left(t_{1/2},T\right) \end{array}$$

$$\begin{array}{l} \bar{\theta }∼Normal\left(0,1\right) \end{array}$$

$$\begin{array}{l} \bar{\beta }∼Normal\left(0,0.25\right) \end{array}$$

$$\begin{array}{l} \sigma_{\theta }∼Exponential\left(1\right) \end{array}$$

$$\begin{array}{l} \sigma_{\beta }∼Exponential\left(1\right) \end{array}$$

$$\begin{array}{l} Rho∼LJKCorr\left(4\right) \end{array}$$

$$\begin{array}{l} V=g\left(t_{1/2},v,T\right) \end{array}$$

$$\begin{array}{l} t_{1/2}∼logNormal\left(log\left(0.25\right),0.25\right) \end{array}$$

$$\begin{array}{l} v∼Exponential\left(20\right) \end{array}$$

All notation is the same as defined above with the addition of $u_{i}$ is now determined by the sum of the product of $dmX_{i}$ and $\theta_{reg\left[i\right]}$, and $X_{i}$ and $\beta_{reg\left[i\right]}$, the latter of which is the direct effect slope that varies per regime. $\theta_{reg\left[i\right]}$ and $\beta_{reg\left[i\right]}$ now have a joint multivariate Gaussian distribution centered on $\bar{\theta }$ and $\bar{\beta }$ with correlation matrix *Rho* and standard deviation for the optima of $\sigma_{\theta }$ and for the slopes $\sigma_{\beta }.$ The prior for *Rho* is the Lewandowski-Kurowicka-Joe distribution, a standard prior for correlation matrices (Lewandowski et al. 2009).

Because of difficulties in exploring the posterior distribution of multilevel models, reparametrizing the model to extract the hyperparameters (e.g., $\bar{\theta }$) from their role as priors can aid in convergence time and improve performance (McElreath 2020; Stan Development Team 2024a). For multilevel models, one approach is switching to a non-centered parameterization (Papaspiliopoulos et al. 2007) to separate parameters. Both the standard and non-centered parameterizations are included in the package, and researchers will need to determine the most efficient model for their own dataset. All mathematical model definitions for the implemented models are included in the Supplementary Materials.

#### Measurement error.

Ignoring measurement error in the predictor and response variables can lead to decreased precision and for the predictors, bias in the estimated regression slopes (Fuller 1987; Hansen and Bartoszek 2012; Silvestro et al. 2015). While *Slouch* and various other approaches include the ability to account for measurement error if quantified as standard errors in either response trait or continuous predictors based on derivations presented in Hansen and Bartoszek (2012)*, Blouch* incorporates measurement error using a Bayesian approach by treating the true quantities as missing data to be estimated (Clayton 1992; Richardson and Gilks 1993).

#### Model comparison.

*Blouch* allows the out-of-sample predictive accuracy of a fitted model to be evaluated using standard Bayesian approaches based on either approximate Leave-One-Out Cross-Validation or Information Criterion by calculating the pointwise log-likelihood values and returning them in the posterior (see Bürkner et al. 2021 for more on this calculation). This includes Pareto-Smoothed Importance Sampling (PSIS), which is an efficient approximation of Leave-One-Out Cross-Validation (Vehtari et al. 2017), and Widely Applicable Information Criterion (WAIC), which converges on cross-validation given increasing sample size but differs in that it approximates K-L divergence, the additional uncertainty introduced by using probabilities from one distribution to describe another distribution (Watanabe 2010). Both can be calculated using the R package *loo* (Vehtari et al. 2024) using the *Blouch* output and both give a way to estimate the degree of overfitting—i.e., making poor predictions due to learning too much from a sample. In addition, *Blouch* allows for the calculation of Bayes Factors, the ratio of the marginal likelihoods—the probability of the observed data given the model—using the R Package *bridgesampling* (Gronau et al. 2020) (see the Supplementary Materials for extended discussion of both approaches). Bayes Factors were used previously in model selection in Bayesian phylogenetic comparative methods (e.g., Uyeda and Harmon 2014), but work (Lindley 1957; Jeffreys 1998; Lartillot 2023) suggests that the use of Bayes Factors may be conservative in model selection when used with vague priors. Pulling back to the big picture, all methods discussed here (and others such as AIC or BIC) are measures of how well the model will perform in predicting new data—estimates of how well it makes out-of-sample predictions. Thus, they should be used for model comparison, not model selection, as differences between the models may be instructive. It should be noted that if a multilevel model is best at prediction over a non-multilevel model, this result is based on how the data is structured—the sample size, the number of optima, etc.—differences between models do not automatically correspond to a biological meaning. This does not mean that in all cases there is no biological meaning for a multilevel model being preferred—it just depends on the biological context. In addition, prediction is not the same as causal inference, and models that are best at prediction may be highly confounded (see McElreath 2020 for an exploration of this point). Finally, the use of predictive accuracy for model comparison in phylogenetic comparative methods is highly complex and unresolved due to the structure of the data (Roberts et al. 2017). Here, I use Pareto-Smoothed Importance Sampling and Bayes Factors to compare models, the former because it also provides a metric for assessing the reliability of the approximation in the form of Pareto *k* diagnostics (see Supplemetary Material; Vehtari et al. 2017), the latter based on their previous use in similar Bayesian phylogenetic comparative methods (Uyeda and Harmon 2014; Uyeda et al. 2017)

#### Prior and posterior predictive checks.

Prior predictive checks generate predictions from the model using only the prior distribution(s) in order to assess whether the priors are appropriate—they are equivalent to running the model without data (Gabry et al. 2019). Posterior predictive checks generate data according to the posterior predictive distribution and compare it to the observed data to assess the fit of the model (Gabry et al. 2019). *Blouch* includes Stan functions to run prior and posterior predictive checks for each of the included models and their use is demonstrated below in the simulation and empirical examples.

### Details

The R package *RStan* (Stan Development Team 2024b) provides the R interface between R and Stan. *Blouch* builds on the R code from *Slouch* (Hansen 1997; Hansen et al. 2008; Kopperud et al. 2020). Basic phylogenetic data formatting is accomplished by the R package *treeplyr* (Uyeda and Harmon 2023), as well as various functions from the R package *ape* (Paradis et al. 2004) and *geiger* (Harmon et al. 2007) as part of the data setup step. *Blouch* provides a convenience R function set.converge.regimes to manually place regimes on a given phylogeny, which labels internal nodes and adds tip regime information to the dataset.

*Blouch* contains a series of different Stan functions that can be run depending on whether there are direct effect or adaptive predictors, multi-optima models, or combinations between the models, and R helper functions to format the data for Stan—decisions on which model to run should be made based on the research question and biological hypotheses about relationships between the factors. Supplementary Table S1 introduces all user functions included in *Blouch* v1.0.

*Blouch*, including R functions, functions written in the probabilistic programming language Stan, and vignettes demonstrating its functionality, is available at GitHub.com. Data files used in the empirical analyses are included in the *Blouch* package, but they are also available in the Dryad data repository.

### Validation

I validated all models on simulated data and report diagnostics in the Supplementary Material and all results can be replicated using code available on GitHub.com. To summarize, all *Blouch* models were able to recover true parameter values with reasonable accuracy in simulated data with half-lives varying from 0.1 to 0.25 to 0.75—from relatively fast adaptation to approaching Brownian motion—and across a range of regime and predictor numbers.

### Simulation Example

To demonstrate the workflow of *Blouch*, a 100-tip tree was created by randomly subsampling from the 301 tip ultrametric molecular primate phylogeny from 10kTrees (consensus tree from version 3; Arnold et al. 2010), which was then scaled to unit length. This methodology follows that of Cressler et al. (2015), who argued that an approach based on a known phylogeny will provide simulated trees that are more realistic than those generated using a pure birth model. Four evolutionary regimes were painted on this tree (Supplementary Fig. S1a), with θ=(1,2,3,4), and β=(0.75,0.5,0.35,0.25). Half-life was set at 10% of tree height, which is a reasonably fast evolution, with *v* at 0.01 as discussed above. As this example will be of the multi-optima adaptive model with varying effects, data for the predictor (*X*) were generated using the fastBM function from the R package *phytools* (Revell 2011) setting an instantaneous variance of the Brownian-motion process ($\sigma^{2}$) to 1. Data for the response (*Y*) was generated using a generative model based on the same model *Blouch* uses to estimate the parameters conditional on the simulated values of *X* and the known parameter values. Measurement error was added to both the response and predictor variables by simulating from a normal distribution centered on 0 with a standard deviation of 0.01, which was added to the data, and the same value (0.01) inputted into *Blouch* as the estimated measurement error for each species value. Two different models were fit to this simulated dataset: a multilevel multi-optima adaptive model with varying effects and a non-multilevel version of this model. Priors were set following the guidelines discussed above, and the Supplementary Materials provides full prior specifications.

I ran 2 independent chains for 4000 generations for each model. As autocorrelation within chains increases uncertainty in the parameter estimates, the effective sample size statistic (*n_eff*) is an estimate of the number of independent draws within a chain that would lead to the same expected precision as the current estimate (Carpenter et al. 2017). It has been suggested that *n_eff* could be as low as 10, but 100 should suffice for many purposes (Gelman et al. 2013). In addition to indicating the convergence of chains for each parameter visually using traceplots, convergence was quantified using the potential scale reduction statistic, $\hat{R}$, which will be close to 1.0 when the chains have converged to the same stationary distribution (Carpenter et al. 2017) and less than 1.1 has been suggested as an appropriate cutoff (Gelman et al. 2013). Models were compared using Pareto-smoothed Importance Sampling and Bayes Factors.

### Empirical Example

**Figure 1.**
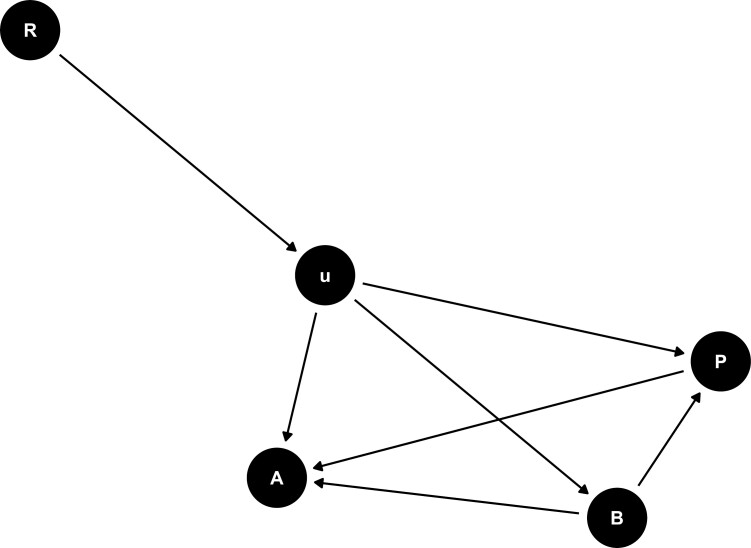
Directed Acyclic Graph (DAG) of the hypothesized relationship between the variables in this analysis. Here, posterior skull length (P), a proxy for body size, is directly influencing antler volume (A), and both P and A are directly influenced by breeding group size (B). In addition, all 3 variables are influenced by unobserved confounds (u), with the phylogenetic relationships among the species (R), influencing u.

Tsuboi et al. (2024) collected an expansive dataset on antler size and body size and generated a new Cervidae phylogeny to test the hypothesis of Gould (1973) that the elaborate antlers of the Irish Elk were the result of a positive allometric relationship with body size. I supplemented this dataset with data on Cervidae breeding group size from Plard et al. (2011) and Clutton-Brock et al. (1980) to test the hypothesis that antler size is influenced by sexual selection by using breeding group size as a predictor of antler size. As formulated by Clutton-Brock et al. (1980), the specific hypothesis is that the relatively larger antlers of larger-bodied deer are the result of more intense sexual selection that comes along with their tendency to live in larger breeding groups. Following Tsuboi et al. (2024), the relationship between posterior skull length, a proxy for body size, and antler volume is hypothesized to be allometric, and thus, the direct effect model is a reasonable choice. Following Hansen (2014), as sexual selection would likely not cause an immediate change in either body size or antler volume, the multi-optima model is indicated. The hypothesized causal relationship between these 3 variables is shown in the directed acyclic graph (DAG; Fig. 1).

Thus, to estimate the direct causal effect of breeding group size on antler volume, I controlled for confounding from posterior skull length and phylogeny as indicated by the DAG by stratifying by these factors. I reduced the phylogeny of Tsuboi et al. (2024) from their original 46 species to the 30 species that had matching breeding group size data and scaled this to unit height. Regimes were reconstructed on the phylogeny using the Ancestral Character Estimation (ace) function from the R package *ape* (Paradis et al. 2004), which produces the scaled likelihoods of ancestral states for each node of the phylogeny (Supplementary Fig. S1b; Pagel 1994). I chose the rate model for the transition rate matrix from 3 possibilities: equal rates, symmetric, and all rates different—with model selection performed using AIC. While using a technique like Stochastic Character Mapping might be preferable to include uncertainty in regime placement (Nielsen 2002; Huelsenbeck et al. 2003) for this simple example, nodes were assigned the maximum likely character state estimated from the ace function, using the equal rates transition matrix, which had the lowest AIC score.

Based on a plot of the data, I tested whether a varying intercepts and varying slopes model (varying effects) might be the most appropriate, fitting both the multilevel multi-optima direct effects model with varying effects and the non-multilevel version of this model. I also fit the multilevel multi-optima direct effects model with varying intercepts and the standard multi-optima direct effects model (Grabowski et al. 2023a), as was done in previous analyses (Bartoszek et al. 2012; Hansen 2014). Neither of the latter two models allow for varying slopes, and I use this example to explore model comparison in *Blouch*.

Priors were set following the guidelines discussed above, and the Supplementary Materials provide the full prior specifications. I ran 2 independent chains for 4000 generations for each model and assessed convergence following the approach used for the simulation example above. Models were compared using Pareto-smoothed Importance Sampling and Bayes Factors.

All *X*/predictor values for the simulations and empirical analyses were mean centered so that the intercept represents the average interspecific *Y* value at the average interspecific *X* value (Pélabon et al. 2014). For the Cervidae analyses, this would translate to mean interspecific species antler volume at the mean interspecific species posterior skull length.

## Results

### Simulation Results

**Figure 2.**
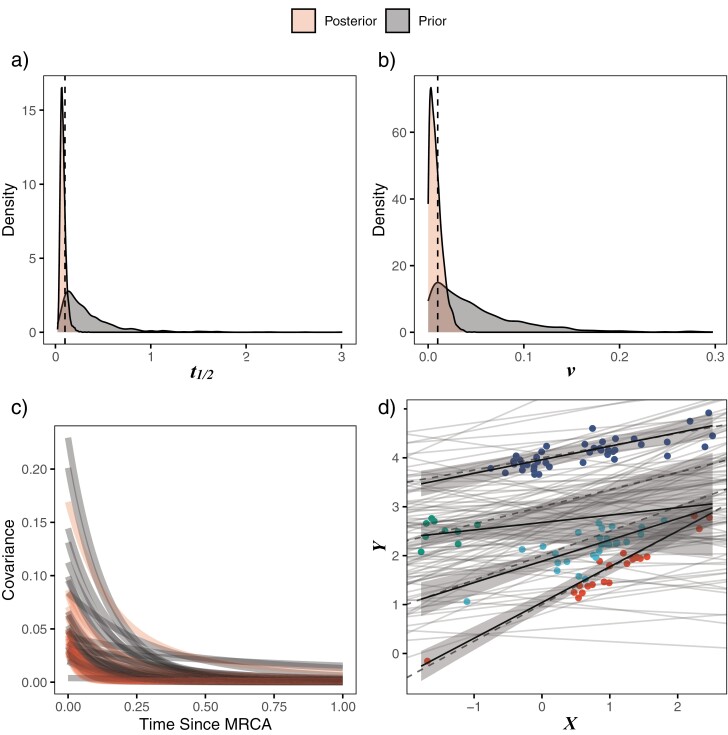
Results for the simulated dataset using the multi-optima adaptive model with varying effects include color-coded prior versus posterior plots for (a) Phylogenetic half-life (*t*_*1/2*_); (b) Stationary variance (*v*); (c) Covariance between residuals from the regression as a reflection of time since MRCA; and (d) Posterior predicted means (black lines) and 89% compatibility intervals (light gray region) for the optimal intercepts and slopes. Species values for each regime are shown in colored circles matching legend colors in Supplementary Figure S1a, with prior predictive simulation results for the intercept and slope shown in light gray lines. The dotted lines are the true values of the parameters.

All parameters for all models had effective sample sizes greater than 1000, suggesting the chains were run at an appropriate length (Supplementary Tables S2 and S3). Along with convergence indicated visually using traceplots (Supplementary Fig. S2), $\hat{R}$ was 1.0 for all parameters (Supplementary Tables S2 and S3). Results (Fig. 2; Supplementary Fig. S3; Supplementary Tables S2 and S3) show good performance at recovering true parameter values, and comparisons of the prior and posterior distributions indicate that the models extract a good deal of information from the data. This includes the estimated covariance between residuals from the regression, which quickly decreases with time from the most recent common ancestor as expected (MRCA; Fig. 2c). The largest deviations in the estimated intercept and slope parameters from the true values are seen in the group with the smallest sample size and least spread along the *X* axis—the second regression line from the top in Figure 2d. This result is expected based on the nature of the sample, but it is notable that the 89% compatibility interval still includes the true values, and the interval expands as it moves away from the data as it should. Model comparisons performed using Pareto-smoothed Importance Sampling show that both models (Supplementary Table S4; Supplementary Fig. S4) produce reasonable Pareto shape values (*k* ~<0.7), indicating that the approximations are reliable (Vehtari et al. 2017), but there is no substantial difference between these two models in their predictive performance (95% CI of the difference between expected log pointwise predictive densities: −0.9–1.3). The two species with consistently high Pareto shape values across the models are *Daubentonia madagascariensis*, the sole member of the lemur family included in the phylogeny, and *Tarsius bancanus,* the only tarsier included—both are distinct due to their long period of independent evolution (first long blue and red branches from the top in Supplementary Fig. S1a), and their distinct nature undoubtably leads to difficulties in predicting their values. On the other hand, Bayes Factors show that the varying effects model is best supported with a Bayes Factor of 63.8 compared to the multilevel version of the same model. The best model results using Bayes Factors are reported in the main text, with other model results reported in the Supplementary Material.

Prior predictive checks show a generally reasonable fit between the data and data generated from the priors, though a few larger true values suggest using the larger scale on some priors may be warranted (Supplementary Fig. S5a). Posterior predictive checks show that the model is well fit as it generates data that are a close approximation of the true dataset (Supplementary Fig. S5b).

### Empirical Results

Because of difficulties in exploring the posterior for the multilevel models, I used the non-centered version of both, which are included as part of the *Blouch* package (Supplementary Table S1). All parameters had effective sample sizes greater than 1000, suggesting the chains were run at an appropriate length (Table 1; Supplementary Tables S5–S7). Along with convergence indicated visually using traceplots (Supplementary Fig. S6), $\hat{R}$ was 1.0 for all parameters (Table 1; Supplementary Tables S5–S7). Pareto-smoothed Importance Sampling model comparison results suggest that all models are similar in their out-of-sample predictions as the difference between their expected log pointwise predictive density is similar to or less than the standard error of the difference (Supplementary Table S8). However, a few high values of the Pareto *k* diagnostic for all models suggest that these results are not reliable (Supplementary Fig. S7). Bayes factor model comparisons found the multi-optima direct effect model with varying effects was best supported with a Bayes Factor of 9.4 over the standard multi-optima direct effect model, 14.4 over the multilevel multi-optima direct effect model with varying effects, and 55.0 over the multilevel multi-optima model with varying intercepts. The best model results using Bayes Factors are reported in the main text, with other model results reported in the Supplementary Material. Prior predictive checks show a reasonable fit between the data and data generated from the priors for the best model, suggesting that the priors are appropriate (Supplementary Fig. S5c). Posterior predictive checks show that the model is well fit as it generates data that are a close approximation of the true dataset (Supplementary Fig. S5d).

**Table 1.** Marginal posterior distribution of the parameters from the multi-optima direct effect model with varying effects for the Cervidae dataset including breeding group size as a predictor.

Parameter	Mean (95% CI)	SD	*n_eff*	$\hat{R}$
*t*_*1/2*_	0.24 (0.15–0.39)	0.06	3820	1
*v*	0.2 (0.1–0.36)	0.07	2751	1
$\theta_{1}$	0.15 (−0.91–1.18)	0.53	1982	1
$\theta_{2}$	0.26 (−0.1–0.63)	0.18	3906	1
$\theta_{3}$	0.54 (0.07–1.02)	0.24	2840	1
$\beta_{1}$	7.25 (5.59–8.82)	0.83	1958	1
$\beta_{2}$	4.46 (3.38–5.64)	0.58	3925	1
$\beta_{3}$	4.61 (3.45–5.85)	0.61	2641	1

Results include mean and 95% compatibility interval (CI), the standard deviation of the mean (SD), the effective sample size statistic (*n_eff*), and the potential scale reduction statistic, $\hat{R}.$ Estimated parameters include the phylogenetic half-life (*t*_*1/2*_) [in units of tree height], the stationary variance (*v*) [in units of squared trait units (log Antler Volume (l)) per unit tree height], the estimated optima/intercepts for increasing breeding group sizes ($\theta_{1-3}$)) [in units of log Antler Volume], and the corresponding slopes ($\beta_{1-3}$)) [in units of log Antler Volume per unit change in log Posterior Skull Length (cm)].

**Figure 3.**
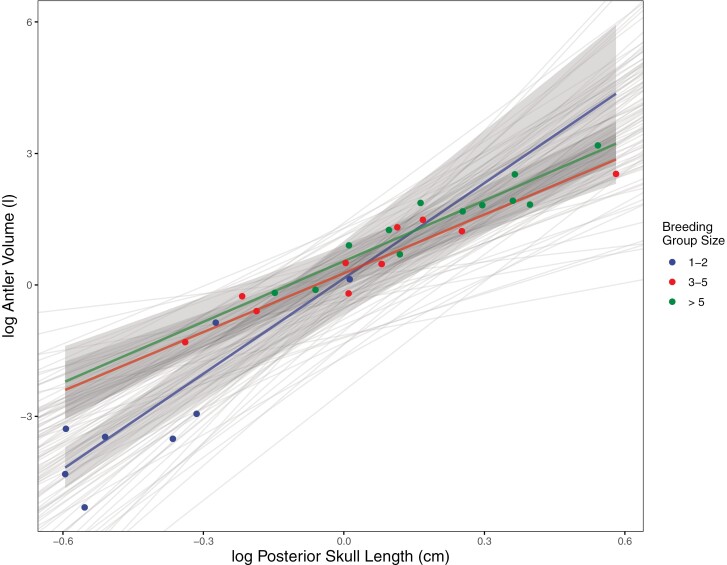
Antler volume as a function of breeding group size stratifying for posterior skull length and phylogeny. Regression lines show posterior means and match the key on the right, with a shaded area showing the 89% compatibility interval of the means, and with prior predictive simulation results for the intercept and slope shown in light gray lines. The 2 larger breeding group sizes (“3-5” and “>5”) have similar slopes, 4.5 ± 0.6 and 4.6 ± 0.6, respectively, while the smallest group size (“1-2”) has a slope of 7.3 ± 0.8.

Results for the best-supported model (Supplementary Fig. S8; Table 1; see also Supplementary Figs. S9–S11) show the 95% compatibility interval for $t_{1/2}$ does not contain 0, and the uppermost limit is below tree height (95% CI: 0.15–0.39), suggesting antler volume adapts to breeding group size in a pattern that differs from instantaneous adaptation and Brownian motion. While the two larger breeding group sizes (“3-5” and “>5”) have similar slopes, 4.5 ± 0.6 and 4.6 ± 0.6, respectively, the smallest group size (“1-2”) has a slope of 7.3 ± 0.8 (Fig. 3). The posterior difference (contrast) between the smallest and medium size group slopes is 2.8 (95% CI: 0.7–4.7), reliably above 0.

## Discussion

Here, I introduced *Blouch*, a Bayesian approach to testing adaptive hypotheses and estimating the evolutionary relationships among traits. Validation results (see Supplementary Material) show good recovery across all included models of true parameter values across a range of half-lives, with increasing compatibility intervals around estimated parameters given increasing half-lives as expected (Hansen 1997; Hansen et al. 2008). Simulation results show good recovery of true parameter values using multi-optima adaptive models with varying effects (Fig. 2; Supplementary Fig. S3; Supplementary Tables S2–S3).

For the empirical Cervidae data, the multi-optima direct effect model with varying effects was the best-supported model using Bayes Factors. Based on a phylogeny height of 14 Ma, results show that antler volume adapts to breeding group size in a pattern that differs from instantaneous adaptation and Brownian motion, with a mean of 3.4 Ma [95% CI: 2.1–5.5]. This is a relatively fast adaptation and might reflect strong sexual selection in this group. For the 3 breeding group sizes, the smallest had a substantially steeper slope than either of the two larger sizes, contrary to previous findings (e.g., Clutton-Brock et al. 1980). Dividing the slope by 3 to account for the dimensionality of volume relative to length shows that all breeding groups have strong positive allometry, with the smallest 2.4 and both larger groups about 1.5 (see also Gould 1973; Plard et al. 2011). These results mean that doubling body size predicts a quintupling of antler size in the smallest group but only a tripling in the larger groups. These findings may paint a different picture of sexual selection and antler size in the Cervidae when compared to previous analyses.

Larger-bodied species are hypothesized to have larger breeding groups than smaller-bodied species and evolve larger antlers relative to body size because of more intense sexual selection (Clutton-Brock et al. 1980). This result was supported using both non-phylogenetic methods (Clutton-Brock et al. 1980), accounting for phylogeny by assuming a Brownian-motion process (Plard et al. 2010), and an Ornstein-Uhlenbeck process (Bartoszek et al. 2012; Hansen 2014), with all showing relative antler size increased in species with larger group sizes. While not directly comparable due to the current analysis using an updated phylogeny and posterior skull length as a proxy for body size rather than error-prone body size estimates used previously (discussed in Tsuboi et al. (2024)), note that the most relevant study, Hansen (2014), used an approach (*Slouch*), which currently only allows intercepts, not slopes, to vary across groups.

The current findings suggest that species in the smallest group size evolve relatively larger antler size with increases in body size. Thus, species in smaller breeding group sizes may be undergoing a different magnitude or type of sexual selection when compared to larger groups. For example, antler size in breeding group sizes of 1–2 individuals may be more strongly influenced by female preference rather than male-male competition, and differences among group sizes may be complex (Wong and Candolin 2005). Alternatively, larger-bodied deer in the smallest group may require relatively larger antlers if inter-male competition increases at a different rate with increasing body size—relatively larger antlers may offer more protection against injury required at a larger size (Clutton-Brock et al. 1980). The inclusion of breeding group size as a predictor and allowing variation in intercepts and slopes across groups may also explain the previous finding that the relationship between log-transformed antler size and body mass is quadratic, rather than linear (Lemaître et al. 2014). While it was suggested that larger-bodied males have relatively smaller antlers than expected from a linear allometric relationship, which could be due to allocating energy away from growth and reproduction and toward maintenance, a different scaling relationship in the smallest breeding groups provides an alternative explanation (Fig. 3) (see also Lemaître et al. 2015; Packard 2015).

These results should be taken with caution for several reasons. First, unlike Bayes Factors, Pareto-smoothed Importance Sampling results suggest all tested models are making similarly (in)accurate predictions. This latter result is likely because there are only a small number of observations in some of the breeding groups (7 species in size 1–2; 10 species in size 3–5; 13 species in size > 5) and leaving out one observation changes the posterior too much to give an accurate prediction of its value—as reflected by the large Pareto *k* diagnostics (Supplementary Fig. S7). In addition, placing breeding groups into qualitative regimes is crude, and their reconstruction on the phylogeny is likely inaccurate, as previously noted (Hansen 2014). The higher slope in the smallest breeding group size is driven by two species—the relatively large antlers in *Muntiacus vuquangensis* and the large antlers and large posterior skull length of *Axis kuhlii* (Fig. 3). Given more or finer-level information on breeding group size from a larger number of Cervidae species would thus be informative. Another option would be robust regression (Gelman et al. 2013)—using a likelihood function that is less sensitive to outliers that are often present in small sample sizes. These can have a greater impact on the shape of the posterior compared to other points and can be minimized using this technique.

This empirical example naturally brings up the issue of sample size required for *Blouch* and other Bayesian phylogenetic comparative methods. Thirty species as in the dataset used here is generally considered small for phylogenetic analyses (Hansen et al. 2008; explored in Bartoszek et al. 2023). However, using a Bayesian approach allows the determination of how much information the data contains about the parameter of interest—if the model extracts very little information from the data, the posterior distribution will resemble the prior. Additional diagnostics such as those used here (i.e., Pareto shape values) provide further feedback about both the sample and model. As a general answer, the question of which model to use comes down to what hypotheses or research questions one is trying to test. If the research question is: “Is there a different relationship between antler size and body size for different breeding group sizes?” a model incorporating both phylogeny, adaptation, allometric relationships, and varying effects is consistent with this question. As argued previously (see Grabowski et al. 2023b), it is always better to use a method that fits the biological question at hand, rather than a simpler and more tractable model that does not.

## Supplementary Material

Data available from the Dryad Digital Repository: https://dx.doi.org/10.5061/dryad.rv15dv4dx.

## Data Availability

*Blouch* and vignettes showing its usage are available on GitHub.com (https://github.com/mark-grabowski/blouch). Complete testing and validation code and results are available at the *Blouch* project site, https://github.com/mark-grabowski/blouch-project.
